# Blood count reference intervals for the Brazilian adult population: National Health Survey

**DOI:** 10.1590/1980-549720230004.supl.1

**Published:** 2023-04-21

**Authors:** Ana Carolina Micheletti Gomide Nogueira de Sá, Nydia Strachman Bacal, Crizian Saar Gomes, Tércia Moreira Ribeiro da Silva, Renata Patrícia Fonseca Gonçalves, Deborah Carvalho Malta

**Affiliations:** I Universidade Federal de Minas Gerais, School of Nursing, Graduate Program in Nursing – Belo Horizonte (MG), Brazil.; II Centro de Hematologia de São Paulo. Clinical Pathology, Flow Cytometry sector Hospital Israelita Albert Einstein – São Paulo (SP), Brazil.; III Universidade Federal de Minas Gerais, School of Medicine, Graduate Program in Public Health – Belo Horizonte (MG), Brazil.; IV Universidade Federal de Minas Gerais, School of Nursing, Department of Maternal and Child Nursing and Public Health – Belo Horizonte (MG), Brazil.; V Universidade Federal dos Vales do Jequitinhonha e Mucuri, Department of Nursing, Graduate Program in Health Education – Diamantina (MG), Brazil.

**Keywords:** Health surveys, Reference values, Blood cell count, Leukocytes, Brazil

## Abstract

**Objective:**

To estimate the reference intervals (RIs) of complete blood count parameters in the Brazilian adult population.

**Methods:**

Cross-sectional study, with data from the National Health Survey (*Pesquisa Nacional de Saúde* – PNS), between 2014–2015. The final sample consisted of 2,803 adults. To establish the RIs, exclusion criteria were applied, outliers were removed and partitions were made by gender, age, and race/skin color. The non-parametric method was adopted. Differences were assessed using the Mann Whitney and Kruskal Wallis tests (p≤0.05).

**Results:**

There were statistically significant differences for the following hematological parameters based on gender, red blood cells, hemoglobin, hematocrit, MCH, MCHC, eosinophils and absolute monocytes, neutrophils and platelets (p≤0.05). When analyzed by age, the RIs were statistically different in females for hematocrit, MCV, white blood cells and RDW and in males for red blood cells, white blood cells, eosinophils, mean platelet volume, MCV, RDW, and MCH (p≤0.05). For race/color, there were differences in the RIs for parameters of hemoglobin, MCH, MCHC, white blood cells and mean platelet volume, neutrophils and absolute eosinophils (p≤0.05).

**Conclusion:**

The differences found in the RIs of some in blood count parameters in Brazilian adults reaffirm the importance of having their own laboratory reference standards. The results can support a more accurate interpretation of tests, adequate identification and disease prevention in Brazil.

## INTRODUCTION

Blood count reference intervals (RIs) (red and white series) are important information in clinical practice^1^ for screening blood donors, assessing overall health, establishing effective diagnosis^2^, and managing and treating diseases^1–3^.

Reliable RIs direct the identification of diseases of important magnitude, such as anemia, infections and neoplasms, and contribute to control and prevention^4^. Anemia represents a global health problem; in 2019, it corresponded to 1.8 billion prevalent cases in the world^5^, and in Brazil, according to data from the National Health Survey (*Pesquisa Nacional de Saúde* – PNS), between 2014 and 2015, there was a prevalence of 9.9% in adults and the aged^6^. In 2019, cancer was the second cause of death worldwide (10,079,637), and in Brazil it corresponded to 266,034 deaths. Respiratory tract infections represented the fourth cause of death worldwide (2,493,199), being the third cause in Brazil (88,640)^7^.

However, establishing RIs is a challenge due to the requirement of methodological rigor with the need for a representative sample of the population; care in collection, transport, biochemical and statistical analysis^1,8^. Thus, determining RIs is not a reality in all countries, being restricted to those that conduct population studies^9^.

Furthermore, RIs are influenced by factors such as race, ethnicity, body mass index (BMI)^10^, circadian rhythm, diet, pregnancy, menstrual cycle^11,12^, menopause^11^, physical activity, stress, smoking and use of medications, alcoholic beverages or caffeine^12^. Therefore, it is recommended to determine the RIs for the population in which they will be applied^10,13^, as they reflect the real health conditions^11^.

Even though the importance of having an RIs belonging to the population is recognized, international RIs are adopted in Brazil^1,12^. So far, there is only one study, in which reference values for blood counts in Brazilian adults were calculated using PNS data, using the parametric method^1^.

The population has influences on the RIs values, therefore, advancing in analytical calculation methods can minimize this effect^2^. The application of a single approach to RIs calculation can lead to inaccuracy, and it is recommended to test other methodologies^13^. Thus, it is important to carry out studies using the same PNS database, in which different methods of RIs determination are adopted.

This study analyzed, for the first time, the blood count RIs of Brazilian adults with laboratory data from the PNS using the non-parametric method, and according to the recommendations of Guideline C28-A3^14^, a reference widely adopted by laboratories^13^. In addition, it advanced by expanding the exclusion criteria, including hemogram parameters according to race/color and in the analyses used for partitioning in relation to the study previously carried out in Brazil^1^.

Thus, the present study aimed to estimate the blood count RIs in the Brazilian adult population.

## METHODS

### Study design

Cross-sectional study, with PNS data, between 2014 and 2015.

### Context and data source

The PNS is a household-based survey carried out by the Brazilian Institute of Geography and Statistics (*Instituto Brasileiro de Geografia e Estatística* – IBGE)^15,16^. In the 2013 PNS, 60,202 adults were interviewed. The collection of tests was planned in a subsample of 25% of the census sectors of the research, and carried out between 2014 and 2015, in 8,952 adults^16^. Blood counts were collected at any time of the day in tubes filled with ethylenediaminetetraacetic acid (EDTA). The blood samples were sent to the reference laboratories that ensured the quality control of the Ministry of Health. The samples were examined by an automatic cell analyzer^16^.

Due to the complex sampling design of the PNS and the unequal selection probabilities, weights were calculated by post-stratification procedures. Sampling weights were adopted in all analyses^16^. Further details on the PNS sampling plan, and the procedures for collecting, sending and storing samples are available in other publications^15,16^. The database used is available at: https://www.pns.icict.fiocruz.br/.

### Determination of blood count reference intervals

In order to reduce the factors that can influence RIs^11^, and aiming to reach a healthy population, exclusion criteria based on the literature^1,8,14^ were applied and the criteria adopted in the national study were expanded^1^. The exclusion criteria used, their references and cutoff points^17–21^ are in Supplementary Material 1.

For outlier exclusion, visual inspection and the Tukey method were used. Outliers were identified in interquartile ranges (IQR), considering the first quartile (Q1) lower and the third quartile (Q3) higher, according to the formula: (Q1-(1.5xIQR); Q3+(1.5xIQR). At levels of <Q1-1.5 IQR and/or >Q3+1.5 IQR, outliers were discarded^13^.

The sample was partitioned according to gender, age and race/skin color, using statistical tests^11,14^ and considering the biological conditions that influence RIs^11^.

RIs were estimated considering 95% of healthy individuals^9^, linked to the 2.5 and 97.5^14^ percentiles. Samples of over 120 individuals were used in partitioning by gender and age^14^.

### Participants

Participants were adults aged 18 years old and older. The PNS database, composed of 8,952 individuals, was used. After exclusion procedures and removal of outliers, the final sample consisted of 2,803 participants.

### Variables

The variables included were: sociodemographic and hemogram parameters (red and white series). The complete description of the variables can be found in Supplementary Material 2.

### Statistical analyses

Medians were calculated for reference limits. The lower limit (LL) was linked to the 2.5^th^ percentile and the upper limit (UL) to the 97.5^th^ percentile of the reference population distribution, according to gender, age, and race/color. RIs were estimated using the non-parametric method, which organizes the observations made by size and classifies them considering the lowest r=1 to the highest r=n. LL corresponded to r=0.025 (n+1) and the UL, the ranking position r=0.975 (n+1)^14^.

Data normality was evaluated using the Shapiro Wilk test and differences were assessed using the Mann Withney or Kruskal Wallis tests, with Dun's post-test with Bonferroni correction, with a significance level of 5%.

Analyses were performed using the Data Analysis and Statistical Software (Stata), version 14, and the Software Package for Social Science (SPSS), version 25.0, using the survey module, which considers post-stratification weights.

### Ethical aspects

The PNS was approved by the National Research Ethics Committee of the National Health Council (Opinion 328.159). Adult participation was voluntary, and confidentiality of information was guaranteed^15^.

## RESULTS

RIs for red blood cells (millions/mm^3^), hemoglobin (g/dL), and hematocrit (%) were higher in men (4.3–5.7; median 5.1; 13.2–16.7; median 15.0; 40.4–52.4; median 45.8) than in women (4.0–5.2; median 4.1; 12.0–15.1; median 13.2; 36.8–47.7; median 41.0). The LL of the MCH (pg) were higher in men (26.7–32.3; median 29.8) than in women (26.5–32.3; median 29.6). In males (30.3–34.4; median 32.8), the UL of the MCHC (g/dL) were also higher than in females (30.4–34.1; median 32.6) (p ≤0.05) (Table 1).

**Table 1:** Red blood cell count reference intervals in adults ≥18 years old by gender, National Health Survey, Brazil, 2014–2015.

Tests	Gender	n	Median	Min-Max	LL	UL	*p
Red blood cells (millions/mm^3^)	Male	1,299	5.1	3.6–6.0	4.3	5.8	<0.01
	Female	1,485	4.5	3.6–6.0	4.0	5.2	
Hemoglobin (g/dL)	Male	1,309	15.0	13.0–17.5	13.2	16.7	<0.01
	Female	1,486	13.2	12.0–17.3	12.0	15.1	
Hematocrit (%)	Male	1,297	45.8	37.2–54.0	40.4	52.4	<0.01
	Female	1,485	41.0	34.5–53.3	36.8	47.7	
Mean Corpuscular Volume (fL)	Male	1,253	90.3	78.7–102.8	82.0	101.0	0.5156
	Female	1,425	90.7	79.0–102.9	81.9	100.9	
Mean corpuscular hemoglobin (pg)	Male	1,269	29.8	26.0–33.2	26.7	32.3	0.0031
	Female	1,442	29.6	26.0–33.2	26.5	32.3	
Mean Corpuscular Hemoglobin Concentration (g/dL)	Male	1,241	32.8	30.1–35.2	30.3	34.4	0.0006
	Female	1,376	32.6	30.1–35.2	30.4	34.5	
Red Cell Distribution Width (RDW) (%)	Male	1,258	13.4	11.7–15.6	12.2	15.4	0.2229
	Female	1,422	13.4	11.5–15.6	12.2	15.3	

n: sample; Min-Max: minimum value and maximum value; LL: lower limit (percentile 2.5); UL: upper limit (percentile 97.5).

* Mann Whitney Test.

RIs of eosinophils (mm^3^) and monocytes (mm^3^) were higher in men (17.5–676.4; median 167.5; 52.0–782.0; median 393.6), compared to women (11.7–671.6; median 150.2; 39.6–752.4; median 353.6). RIs of platelets (μl) and neutrophils (mm^3^) were higher in women (145,000–337,000; median 234,000; 887.0–6,429.6; median 3,374.1) than in men (143,000–315,000; median 210,000; 798.0–6,114.3; median 3,143.7) (p≤0.05) (Table 2).

**Table 2:** White series blood count reference intervals in adults ≥ 18 years old, according to gender, National Health Survey, Brazil, 2014-2015.

Tests	Gender	n	Median	Min-Max	LL	UL	p*
White blood cells (mm^3^)	Male	1,184	5,900	1,900–10,900	2,800	9,700	0.0977
	Female	1,308	6,120	1,300–10,800	2,600	9,900	
Absolute neutrophils (mm^3^)	Male	1,173	3,143.7	399.6–7,101.0	798.0	6,114.3	0.0101
	Female	1,283	3,374.1	193.5–7,078.5	887.0	6,429.6	
Absolute eosinophils (mm^3^)	Male	1,060	167.5	0.0–765.4	17.5	676.4	0.0006
	Female	1,246	150.2	0.0–775.0	11.7	671.6	
Absolute basophils (mm^3^)	Male	1,165	23.4	0.0–99.2	0.0	78.0	0.1459
	Female	1,295	22.2	0.0–99.0	0.0	80.5	
Absolute lymphocytes (mm^3^)	Male	1,158	1,996.9	255.6–3,843.0	717.0	3,511.2	0.8142
	Female	1,293	2,002.2	303.4–3,874.2	742.0	3,411.4	
Absolute monocytes (mm^3^)	Male	1,157	393.6	3.1–900.6	52.0	782	<0.01
	Female	1,301	353.6	0.0–896.0	39.6	752.4	
Platelets (μl)	Male	1,183	210,000	105,000–365,000	143,000	315,000	<0.01
	Female	1,353	234,000	126,000–364,000	145,000	337,000	
Mean platelet volume (fL)	Male	962	10.1	7.5–13.2	8.2	12.6	0.0503
	Female	1,136	10.3	7.6–13.2	8.3	12.6	

n: sample; Min-Max: minimum value and maximum value; LL: lower limit (percentile 2.5); UL: upper limit (percentile 97.5).

* Mann Whitney Test.

In men, the RIs of red blood cells (millions/mm^3^) were higher between 18 and 39 years of age (4.4–5.8; median 5.1), compared with the age groups from 40 to 59 years (4.3–5.7; median 5.0) and 60 years old or older (4.2–5.8; median 4.8). RIs of MCV (fL) and MCH (pg) were lower in men between 18 and 39 years old (81.8–100.4; median 89.4; 26.4–32.0; median 29.5) and 40 to 59 years old (82.6–101.2; median 91.4; 26.0–32.2; median 30.0) than those aged 60 years old or older (83.9–102.0; median 92.7; 27.2–38.2; median 30.7) (p≤0.05). The median and UL of RDW (%) were higher in men aged 60 years old and older (12.2–15.5; median 13.5) than in men aged 18 to 39 years (12.4–15.3; median 13.3) and 40 to 59 years old (12.3–15.3; median 13.3) (p≤0.05) (Supplementary Material 3).

In women, there were differences for LL and UL, and the median was lower for hematocrit (%) between 18 and 39 years old (36.9–47.3; median 40.7), when compared to 40 to 59 years old (36.6–48.4; median 41.3) and 60 years old or older (36.5–47.1; median 41.2). MCV's (fL) RIs were lower in women aged 18 to 39 years (81.6–100.9; median 90.5) than those aged 40 to 59 years (82.2–100.7; median 91.2). The LL and median of the RDW (%) were lower in women aged 18 to 39 years (12.1–15.2; median 13.4) than in those aged 40 to 59 years (12.3–15.4; median 13.5) and 60 years old or older (12.3–15.2; median 13.7) (p≤0.05) (Supplementary Material 4).

Men had higher white blood cell counts (mm^3^) between ages 18 and 39 (2,970–9,990; median 6,000), compared with those aged 60 years old and older (2,600–9,400; median 5,640). The LL and median of the eosinophils (mm^3^) were higher in men aged 18 to 39 years (26.1–661.2; median 190.1), compared with men aged 40 to 59 years (16.8–679.8; median 141.6). RIs for platelets (μl) were lower in men with increasing age (18–39 years: 144,000–314,000; median 215,000; 40–59 years: 143,000–322,000; median 215,000; 60 years old and older: 138,000–306,000; median 203,000; p≤0.05) (Supplementary Material 5).

In women, there were differences for RIs of white blood cells (mm^3^); the median and LL were more prominent between 18 and 39 years of age (2,600-10,000; median 6,300) and 40 to 59 years (2,800–9,800; median 5,800) than at 60 years old and older (2,000–9,800; median 5,500) (p≤0.05) (Supplementary Material 6).

Hemoglobin (g/dL) and MCH (pg) RIs were higher in white men (13.3–16.8; median 15.1; 27.0–32.4; median 29.9) than in brown ones (13.1–17.3; median 14.8; 26.5–32.2; median 29.7). MCH medians were lower in black men (29.3 pg) than in white men (29.9 pg). The UL and median of the MCHC (g/dL) were slightly higher in white men (30.4–34.6; median 32.8) than in brown (30.3–34.4; median 32.6) and black men (30.5–34.3; median 32.8) (p≤0.05). RIs of eosinophils (mm^3^) were lower in white (17.1–648.0; median 151.2) than in black (38.5–678.5; median 230.1) and brown men (17.5 –688.1; median 194.0) (p≤0.05) (Supplementary Material 7).

RIs of hemoglobin (g/dL) and MCH (pg) were higher in white women (12.1–15.1; median 13.4; 27.0–32.3; median 29.8) than in brown (12.0–15.0; median 13.2; 26.4–32.2; median 29.4) and black ones (12.0–14.8; median 13.1; 26.3–31.8; median 28.9). The MCV (fL) median and LL was more prominent in white women (82.7–100.4; median 91.0) than in black women (82.4–100.9; median 89.5). The UL of the MCHC (g/dL) was higher in white (30.4–34.2; median 32.7) than in brown (30.3–34.0; median 32.5) and black women (30.5–33.7; median 32.4). White women (2,900–10,000; median 6,300) had higher median and LL values for white blood cells (mm^3^) than brown (2,500–9,700; median 5,900) and black women (2,650–10,100; median 5,600). In brown (median 3,129) and black (median 2,866) women, median neutrophil (mm^3^) were higher than in white women (median 2,597). In brown women (9.3–694.4; median 150.0), the median and UL of eosinophils (mm^3^) were higher than in white women (14.2–660.0; median 140.6). The median and LI of mean platelet volume (fL) was higher in white women (8.4–12.7; median 10.4) than in brown (8.2–12.3; median 10.2) and black women (8.3–12.9; median 10.2) (p≤0.05) (Supplementary Material 7).

## DISCUSSION

In this study, blood count RIs of Brazilian adults was estimated by PNS tests, using the non-parametric method. Statistically significant differences were observed for some components of the red and white series blood count when analyzed according to gender, age, and race/color. RIs were calculated using a methodology that had not yet been tested, following recommendations in the literature, in order to obtain increasingly accurate and reliable values^13^. Therefore, it differed from the only existing national study in which reference values were calculated for Brazilian adults, by using a non-parametric approach and also by the tests applied for sample stratification, application of the Tukey method to remove outliers and the expansion of the exclusion criteria of the study by Rosenfeld et al.^1^.

The non-parametric methodology adopted here is in line with studies carried out in Ghana^3^, Canada^8^, India^9^, Kenya^10^, and Korea^22^. This method is recommended due to many analytes not having a normal distribution, and because it is simpler, depending only on the classifications of the reference data arranged in increasing order of size^14^. The literature describes that the results found in parametric and non-parametric methods are usually similar^14^. As can be seen from the median values found in this study according to gender and the mean values identified in the national study for red and white blood cells were 5.0 million/mm^3^ and 6,142 mm^3^ in men and 4.5 million/mm^3^ and 6,426 mm^3^ in women, respectively^1^.

For selection of healthy individuals, exclusions were defined according to Guideline C28-A3^14^ and based on studies^1,8–10,23^. The procedures adopted here for removing outliers were used in studies in Canada^8^ and Oman^24^. Tukey's method was used because it is more useful and indicated in the presence of more than one outlier^13^, which occurred in this study, and visual inspection because it is considered effective^13^.

Considering partitioning as a power tool for diagnosing RIs^7^, this investigation used statistical tests to verify its need, as well as in other studies^22–25^. The partitioning adopted is set out in Guideline C28-A3^15^, and the physiological changes of adulthood were also considered^11^. When calculating RIs, the physiological aspects are as important as the statistical ones^11,14^, as RIs differ in children, adults, the aged, men and women; in puberty, pregnancy and menopause, due to organic changes throughout life^11^.

The highest RIs for red blood cells, hemoglobin, and hematocrit identified in men, compared to women, and higher RIs for platelets in women than in men, were also found in Ghana^3^, Oman^24^, and Brazil^1^. In this study, as well as in an investigation carried out in Morocco^25^, higher RIs of MCH and MCHC were observed in men compared to women. Differences between genders for erythrocytes, hemoglobin, hematocrit, MCH, MCHC, platelets, and platelet volume^1^ have been documented, which can be explained by the effect of menstruation and the increased demand for iron^3,23^, hormonal influences of androgen^1,3,8^ and the way in which erythropoiesis and megakaryopoiesis are regulated in men and women^3^.

Our findings agree with studies from Brazil^1^, Canada^8^, Korea^23^, and Morocco^25^, in which neutrophil levels were lower in men than in women, possibly due to impacts related to sexual development on immunity, in which estrogen stimulates an immune response, and some androgen hormones, such as testosterone, suppress the response to infection, with neutrophil levels being highest in females during puberty and adulthood^8,23^.

Studies carried out in Brazil^1^, Canada^8^, and China^22^ found differences in monocyte RIs according to gender^8,22^, being higher in men, in line with our results. It is known that men can have higher monocyte counts than women, however the physiological and clinical aspects still need to be elucidated^8^. Higher eosinophil RIs in men than in women were also found in studies in Korea^23^, Morocco^25^, China^22^, Ghana^3^, and Brazil^1^. However, research is needed to investigate the reasons for the biological fluctuations of these leukocyte parameters in specific populations from different locations according to gender^9^.

The subtle differences found in hematocrit RIs in women, which are higher after 40 years of age, compared to 18 to 39 years of age, are justified by the variability of this parameter, which is similar throughout life^8^. Slight increase in hematocrit with age was found in Chinese adults. This finding is consistent with lower levels before menopause and higher levels after this period^2^. Red blood cell values tended to decrease with age in males, as in Chinese adults^2^. The decrease may be due to the gradual loss of androgen^2^. The lowest RIs from 60 years of age onward in men may be related to nutritional deficiency, occult malignancy, and anemia^2^.

For the RIs of white blood cells, the results found are similar to those of other studies^23,24^, in which a decline was observed with increasing age^8^. The findings reaffirm that two divisions by age could be made for this parameter, as in the Brazilian study^1^, as there were no differences between the two age groups of 18 to 39 and 40 to 59 years. Higher white blood cell counts in young adults of both genders, when compared to lower counts in the aged, reflect the development of the adaptive and acquired immune response as the immune system is more exposed to pathogens and antigens in the environment^8,23^.

In this study, the slight increase in eosinophil UL with age in men may be related to chronic infections in aged people who were not excluded^2^. This finding was present in Chinese adults of both genders^2^. Differences in eosinophil values can be attributed to allergic and parasitic diseases in apparently healthy adults^2^.

Studies in Canada^8^ and Brazil^1^ also identified a slight increase in MCV and RDW throughout life, with small changes in early adulthood, remaining constant after this phase^8^. A study in China identified that aged men and women had higher RIs for RDW ^23^. MCV and RDW provide a classification of erythrocytes based on size and distribution^4^. For MCV values, the LL is approximately 70 fL plus age (in years), and the UL can be obtained by adding 0.6 fL per year to 84 fL beyond the first year of life, until approximately 96 fL is reached in adults^4^, consistent with the findings of this study. RDW is useful in identifying iron deficiency, β-thalassemia trait, inflammatory processes, and chronic infection^4^. It is also a predictor of mortality, and its increase with age is related to organic changes in aging and chronic diseases in this phase^1^.

In this study, significant differences in MCH values with age in men, being higher in the aged, was identified in a previous study^1^. It is noteworthy that the RIs for all age groups in Brazilian men were lower than the international hematrimetric classification (27 to 33.7 pg)^2^. The literature shows regional variations for HCM; in Chinese, RIs did not differ according to age and gender^2^, while in Moroccan men there were differences^25^. Although our study found differences in men, the international classification covers the same RIs values for MCH according to gender and age for adults^2^. In this sense, further investigations are desirable to clarify the implications of these differences in clinical practice.

The lowest platelet count identified in aged Brazilian men is in line with a study in Canada^8^ and was found in men over 40 years of age in Iran^9^. A possible justification is the gradual decline of thrombopoietin, a hormone that regulates the production of platelets in adults. The occurrence of thrombocytopenia is more common in men than in women, and more frequent in the aged^8^.

In this study, RIs was established for 16 hemogram parameters according to race/color. In a previous research, RIs of five parameters were determined, and the values found were close to those described^1^. Although discreet, the differences found according to race/color for RIs of hemoglobin, MCH, MCHC, white blood cells and mean platelet volume, neutrophils and eosinophils, support the need to establish RIs for Brazilians. Differences in the blood count RIs were found in populations from other countries^1–3,8,10,24^, reinforcing the importance of considering geographic and ethnic-racial influences, as they may be related to factors such as genetics, nutrition, socioeconomic, cultural, and lifestyle factors, exposure to allergens, infections and parasitic loads in different locations^3^.

Limitations, such as the possibility of including patients without a previous diagnosis and the failure to obtain samples with 120 individuals for some strata of race/color, must be considered. However, due to the representativeness of the sample, it is emphasized that this study is close to the reality of the health conditions of the Brazilian population. Furthermore, the literature documents that it is possible to establish a RIs of 95% using up to 39 samples^13^. As for the losses attributed to the procedures of exclusion and removal of outliers, this is a conservative bias in clinical practice, as it was possible to estimate the RIs of blood counts for Brazilian adults and predict that the values found were close to the previous national study^1^, taking into account the recommendations to test other methodological approaches^13^. The RIs found here confer reliability and allow the generalization of the findings in a relatively safe way. Differences found in some hemogram parameters in Brazilian adults according to gender, age, and race/color show that there is a need to establish RIs that are adequate for the population. The results show the ethnic-racial influences on the RIs and can support the identification and prevention of diseases, as well as future research to validate the RIs of the Brazilian population, contributing to better interpretation, diagnostic accuracy, and quality of care and treatment offered.
