# Supplementary material for: Blood count reference intervals for the Brazilian adult population: National Health Survey
Source: Rev Bras Epidemiol. 2023 Apr 21;26(Suppl 1):e230004. doi: 10.1590/1980-549720230004.supl.1 (PMC10176730; doi:10.1590/1980-549720230004.supl.1)
Supplement: Supplementary file 1 [file 1980-5497-rbepid-26-suppl1-e230004-suppl1.docx]

**Material Suplementar 1. Critérios de exclusão adotados no estudo. Pesquisa Nacional de Saúde (PNS), Brasil, 2014-2015**

| **Critérios de exclusão** | **Descrição** |
| --- | --- |
| Pessoas com idade abaixo de 18 anos | Autorreferido* |
| Obesidade | Índice de Massa Corporal aferido ( ≥ 30 kg/m^2^ )^17.^ Aferido**. |
| Hipertensão arterial | Autorreferida* e aferida** (≥ 140/90 mmHg)^18^ |
| Doença renal crônica | Taxa de filtração Glomerular (TGF)** calculada pela equação Chronic Kidney Disease Epidemiology Collaboration (< 60 mL/min/1,73m2)^19^ (identificada por creatinina dosada em exame de sangue). |
| Diabetes | Autorreferida e HbA1c** ≥ 6,5%^20^ (identificado por exame de sangue) |
| Hemoglobinopatias | Traço falciforme, persistência da hemoglobina fetal, traço falciforme com hemoglobina C, talassemia menor, suspeita de talassemia maior e menor e ser portador de hemoglobina C** (identificados no exame de sangue) |
| Anemia | Hemoglobina** < 12 g/dL em mulheres e hemoglobina** < 13 g/dL em homens^21^ (identificado por exame de sangue) |
| Adultos sem resultados de exames | Dados faltosos |
| Grávidas | Autorreferido* |
| Tabagismo | Autorreferido* |
| Doença pulmonar obstrutiva crônica | Autorreferido* |
| Câncer | Autorreferido* |
| Doença cardiovascular | Autorreferido* |
| Acidente vascular cerebral; | Autorreferido* |
| Artrite | Autorreferido* |
| Reumatismo | Autorreferido* |

*Dados autorreferidos da PNS. Dados identificador por exames laboratoriais da PNS. Dados faltosos: correspondem a adultos sem resultados de exames na PNS.

**Material Suplementar 2 - Variáveis utilizadas no estudo e suas descrições. Códigos dos indicadores hematológicos no banco de dados da Pesquisa Nacional de Saúde, Brasil, 2014-2015**

| **Variáveis** | **Descrição das variáveis e códigos dos indicadores hematológicos no banco de dados da PNS** |
| --- | --- |
| *Sociodemográficas* | |
| Sexo | Masculino e feminino. |
| Idade (faixa etária em anos) | 18 a 39 anos, 40 a 59 anos e 60 anos ou mais. |
| Raça/cor da pele | Branca, parda e preta. |
| *Série vermelha do hemograma* | |
| Glóbulos vermelhos | Em milhões/mm^3^. Código Z006. |
| Hemoglobina | Em g/dL. Código Z007. |
| Hematócrito | Em %. Código Z008. |
| Volume Corpuscular Médio (VCM) | Em fL. Código Z009. |
| Hemoglobina Corpuscular Média (HCM) | Em pg. Código Z010. |
| Concentração de Hemoglobina Corpuscular Média (CHCM) | Em g/dL. Código Z010a. |
| Amplitude de distribuição dos eritrócitos (RDW) | Em %. Código Z011. |
| *Série branca do hemograma* | |
| Glóbulos Brancos | Em mm^3^. Código Z012. |
| Neutrófilos Absolutos | Em mm^3^. Código Z013. |
| Eosinófilos Absolutos | Em mm^3^. Código Z015. |
| Basófilos Absolutos | Em mm^3^. Código Z017. |
| Linfócitos Absolutos | Em mm^3^. Código Z019. |
| Monócitos Absolutos | Em mm^3^. Código Z021. |
| Plaquetas | Em μl. Código Z023. |
| Volume plaquetário Médio (VPM) | Em fL. Código Z024. |

**Material Suplementar 3 – Intervalos de referência de hemograma série vermelha no sexo masculino (≥ 18 anos) segundo idade, Pesquisa Nacional de Saúde, Brasil, 2014-2015**

| **Exames** | **Idade** | **n** | **Mediana** | **Min-Max** | **LI** | **LS** | **p*** |
| --- | --- | --- | --- | --- | --- | --- | --- |
| Glóbulos vermelhos (milhões/mm^3^) | 18-39^a^ | 637 | 5,1 | 3,6-6,0 | 4,4 | 5,8 | 0,0001 |
|  | 40-59^b^ | 507 | 5,0 | 4,0-6,0 | 4,3 | 5,7 |  |
|  | 60 ou mais^c^ | 155 | 4,8 | 4,0-5,9 | 4,2 | 5,8 |  |
| Hemoglobina (g/dL) | 18-39 | 642 | 15,1 | 13,0-17,5 | 13,3 | 16,7 | 0,821 |
|  | 40-59 | 511 | 15,0 | 13,0-17,5 | 13,3 | 16,8 |  |
|  | 60 ou mais | 156 | 14,7 | 13,0-17,3 | 13,1 | 16,8 |  |
| Hematócrito (%) | 18-39 | 638 | 45,8 | 38,6-54,0 | 40,5 | 52,3 | 0,4868 |
|  | 40-59 | 504 | 45,8 | 38,3-53,8 | 40,5 | 52,5 |  |
|  | 60 ou mais | 155 | 45,7 | 37,2-53,7 | 39,5 | 52,9 |  |
| Volume Corpuscular Médio (fL) | 18-39^a^ | 617 | 89,4 | 78,7-102,8 | 81,8 | 100,4 | 0,0001 |
|  | 40-59^b^ | 496 | 91,4 | 79,0-102,8 | 82,6 | 101,2 |  |
|  | 60 ou mais^c^ | 140 | 92,7 | 78,8-102,8 | 83,9 | 102,0 |  |
| Hemoglobina Corpuscular Média (pg) | 18-39^a^ | 626 | 29,5 | 26,0-33,2 | 26,4 | 32,0 | 0,0001 |
|  | 40-59^b^ | 497 | 30,0 | 26,0-33,2 | 26,8 | 32,4 |  |
|  | 60 ou mais^c^ | 146 | 30,7 | 26,9-33,2 | 27,2 | 32,8 |  |
| Concentração de Hemoglobina Corpuscular Média (g/dL) | 18-39 | 612 | 32,9 | 30,1-35,1 | 30,3 | 34,4 | 0,257 |
|  | 40-59 | 483 | 32,7 | 30,1-35,2 | 30,4 | 34,3 |  |
|  | 60 ou mais | 146 | 32,5 | 30,1-35,0 | 30,2 | 34,6 |  |
| Amplitude de distribuição dos eritrócitos (RDW) (%) | 18-39^a^ | 628 | 13,3 | 11,8-15,6 | 12,4 | 15,3 | 0,0118 |
|  | 40-59^a^ | 492 | 13,3 | 11,7-15,6 | 12,3 | 15,3 |  |
|  | 60 ou mais^b^ | 138 | 13,5 | 11,9-15,6 | 12,2 | 15,5 |  |

n: amostra. Min-Max: valor mínimo e valor máximo. LI: limite inferior (percentil 2,5). LS: Limite superior (percentil 97,5%). *Teste de Kruskal Wallis. a,b,c: letras iguais representam que não houve diferenças estatisticamente significativas entre os grupos (p> 0,05); a,b,c: letras diferentes representam que houve diferenças estatisticamente significativas entre os grupos (p≤ 0,05).

**Material Suplementar 4 – Intervalos de referência de hemograma série vermelha no sexo feminino ( ≥ 18 anos) segundo idade, Pesquisa Nacional de Saúde, Brasil, 2014-2015**

| **Exames** | **Idade** | **n** | **Mediana** | **Min-Max** | **LI** | **LS** | **p*** |
| --- | --- | --- | --- | --- | --- | --- | --- |
| Glóbulos vermelhos (milhões/mm^3^) | 18-39 | 840 | 4,5 | 3,6-6,0 | 4,0 | 5,2 | 0,352 |
|  | 40-59 | 510 | 4,5 | 3,8-5,5 | 4,0 | 5,2 |  |
|  | 60 ou mais | 135 | 4,5 | 4,0-5,6 | 4,0 | 5,3 |  |
| Hemoglobina (g/dL) | 18-39 | 841 | 13,2 | 12,0-17,3 | 12,0 | 14,9 | 0,051 |
|  | 40-59 | 510 | 13,4 | 12,0-17,1 | 12,1 | 15,3 |  |
|  | 60 ou mais | 135 | 13,1 | 12,0-16,6 | 12,0 | 15,2 |  |
| Hematócrito (%) | 18-39^a^ | 839 | 40,7 | 35,4-52,6 | 36,9 | 47,3 | 0,0093 |
|  | 40-59^b^ | 511 | 41,3 | 34,5-53,3 | 36,6 | 48,4 |  |
|  | 60 ou mais^b^ | 135 | 41,2 | 36,0-51,6 | 36,5 | 47,1 |  |
| Volume Corpuscular Médio (fL) | 18-39^a^ | 802 | 90,5 | 79,0-102,8 | 81,6 | 100,9 | 0,0081 |
|  | 40-59^b^ | 494 | 91,2 | 79,1-102,9 | 82,2 | 100,7 |  |
|  | 60 ou mais^a,b^ | 129 | 91,1 | 79,1-102,4 | 81,5 | 101,5 |  |
| Hemoglobina Corpuscular Média (pg) | 18-39 | 810 | 29,5 | 26,0-33,1 | 26,5 | 32,3 | 0,0958 |
|  | 40-59 | 500 | 29,7 | 26,0-33,2 | 26,7 | 32,3 |  |
|  | 60 ou mais | 132 | 29,5 | 26,0-32,4 | 26,5 | 31,9 |  |
| Concentração de Hemoglobina Corpuscular Média (g/dL) | 18-39 | 782 | 32,6 | 30,1-35,2 | 30,4 | 34,0 | 0,9037 |
|  | 40-59 | 472 | 32,6 | 30,1-35,1 | 30,3 | 34,1 |  |
|  | 60 ou mais | 122 | 32,5 | 30,1-34,7 | 30,4 | 34,3 |  |
| Amplitude de distribuição dos eritrócitos (RDW) (%) | 18-39^a^ | 808 | 13,4 | 11,6-15,6 | 12,1 | 15,2 | 0,0001 |
|  | 40-59^b^ | 487 | 13,5 | 11,5-15,6 | 12,3 | 15,4 |  |
|  | 60 ou mais^c^ | 127 | 13,7 | 12,1-15,6 | 12,3 | 15,2 |  |

n: amostra. Min-Max: valor mínimo e valor máximo. LI: limite inferior (percentil 2,5). LS: Limite superior (percentil 97,5%). *Teste de Kruskal Wallis. a,b,c: letras iguais representam que não houve diferenças estatisticamente significativas entre os grupos (p> 0,05); a,b,c: letras diferentes representam que houve diferenças estatisticamente significativas entre os grupos (p≤ 0,05).

**Material Suplementar 5 – Intervalos de referência de hemograma série branca no sexo masculino (≥ 18 anos) segundo idade, Pesquisa Nacional de Saúde, Brasil, 2014-2015**

| **Exames** | **Idade** | **n** | **Mediana** | **Min-Max** | **LI** | **LS** | **p*** |
| --- | --- | --- | --- | --- | --- | --- | --- |
| Glóbulos Brancos (mm^3^) | 18-39^a^ | 587 | 6.000 | 1.900-10.800 | 2.970 | 9.990 | 0,0318 |
|  | 40-59^a,b^ | 463 | 5.800 | 2.300-10.900 | 2.800 | 9.600 |  |
|  | 60 ou mais^b^ | 134 | 5.640 | 2.200-9.600 | 2.600 | 9.400 |  |
| Neutrófilos Absolutos (mm^3^) | 18-39 | 581 | 3.144,7 | 400,0-7.091,0 | 751,6 | 6.103,0 | 0,7167 |
|  | 40-59 | 557 | 3.128,4 | 608,0-7.101,0 | 791,3 | 6.114,3 |  |
|  | 60 ou mais | 135 | 3.031,7 | 640,0-6.999,0 | 1.014,0 | 6.274,4 |  |
| Eosinófilos Absolutos (mm^3^) | 18-39^a^ | 517 | 190,1 | 0,0-765,4 | 26,1 | 661,2 | 0,0089 |
|  | 40-59^b^ | 419 | 141,6 | 0,0-763,8 | 16,8 | 679,8 |  |
|  | 60 ou mais^a,b^ | 124 | 173,4 | 0,0-688,8 | 0,7 | 674,0 |  |
| Basófilos Absolutos (mm^3^) | 18-39 | 576 | 23,4 | 0,0-98,7 | 0,0 | 79,2 | 0,8203 |
|  | 40-59 | 450 | 24,0 | 0,0-99,2 | 0,0 | 75,6 |  |
|  | 60 ou mais | 139 | 23,1 | 0,0-96,9 | 0,0 | 81,4 |  |
| Linfócitos Absolutos (mm^3^) | 18-39 | 576 | 2.083,2 | 365,4-3.769,6 | 725,0 | 3.525,0 | 0,5445 |
|  | 40-59 | 447 | 1.904,0 | 255,6-3.843,0 | 716,8 | 3.488,7 |  |
|  | 60 ou mais | 135 | 1.683,5 | 438,4-3.827,0 | 709,8 | 2.954,6 |  |
| Monócitos Absolutos (mm^3^) | 18-39 | 580 | 413,0 | 8,8-900,6 | 45,6 | 782,6 | 0,1158 |
|  | 40-59 | 443 | 360,8 | 3,1-870,0 | 52,0 | 778,8 |  |
|  | 60 ou mais | 134 | 390,5 | 26,0-868,4 | 85,1 | 782,0 |  |
| Plaquetas (μl) | 18-39^a^ | 594 | 215.000 | 131.000-357.000 | 144.000 | 314.000 | 0,0017 |
|  | 40-59^b^ | 465 | 205.000 | 112.000-359.000 | 143.000 | 322.000 |  |
|  | 60 ou mais^b^ | 124 | 203.000 | 105.000-365.000 | 138.000 | 306.000 |  |
| Volume plaquetário Médio (fL) | 18-39 | 479 | 10,0 | 7,5-13,2 | 8,1 | 12,6 | 0,3789 |
|  | 40-59 | 378 | 10,3 | 7,6-13,1 | 8,1 | 12,6 |  |
|  | 60 ou mais | 105 | 10,2 | 8,1-12,9 | 8,5 | 12,6 |  |

n: amostra. Min-Max: valor mínimo e valor máximo. LI: limite inferior (percentil 2,5). LS: Limite superior (percentil 97,5%). *Teste de Kruskal Wallis. a,b,c: letras iguais representam que não houve diferenças estatisticamente significativas entre os grupos (p> 0,05); a,b,c: letras diferentes representam que houve diferenças estatisticamente significativas entre os grupos (p≤ 0,05).

**Material Suplementar 6 – Intervalos de referência de hemograma série branca no sexo feminino (≥ 18 anos) segundo idade, Pesquisa Nacional de Saúde, Brasil, 2014-2015**

| Glóbulos Brancos (mm^3^) | 18-39^a^ | 738 | 6.300 | 1.300-10.800 | 2.600 | 10.000 | 0,0077 |
| --- | --- | --- | --- | --- | --- | --- | --- |
|  | 40-59^a,a^ | 452 | 5.800 | 1.500-10.500 | 2.800 | 9.580 |  |
|  | 60 ou mais^b^ | 118 | 5.500 | 1.500-10.400 | 2.000 | 9.800 |  |
| Neutrófilos Absolutos (mm^3^) | 18-39 | 726 | 3.610,2 | 194-7.079 | 882 | 6.572,0 | 0,0831 |
|  | 40-59 | 442 | 3.129,5 | 587-7.048 | 972 | 6.035,2 |  |
|  | 60 ou mais | 115 | 2.893,5 | 594-7.046 | 756 | 5.955,0 |  |
| Eosinófilos Absolutos (mm^3^) | 18-39 | 700 | 158,4 | 0-775,0 | 22,2 | 684,0 | 0,0975 |
|  | 40-59 | 428 | 134,4 | 0-773,5 | 9,9 | 627,9 |  |
|  | 60 ou mais | 118 | 148,5 | 0-759,5 | 0,0 | 672,9 |  |
| Basófilos Absolutos (mm^3^) | 18-39 | 738 | 22,0 | 0-99,0 | 0,0 | 82,4 | 0,3006 |
|  | 40-59 | 445 | 22,0 | 0-95,2 | 0,0 | 79,6 |  |
|  | 60 ou mais | 112 | 20,0 | 0-87,5 | 0,0 | 84,0 |  |
| Linfócitos Absolutos (mm^3^) | 18-39 | 732 | 2.025,2 | 303,4-3.829,8 | 607,6 | 3.405,4 | 0,3242 |
|  | 40-59 | 448 | 1.995,0 | 372,0-3.874,2 | 844,2 | 3.617,6 |  |
|  | 60 ou mais | 113 | 1.771,9 | 402,8-3.374,6 | 787,4 | 2.935,8 |  |
| Monócitos Absolutos (mm^3^) | 18-39 | 738 | 357,5 | 0-896,0 | 44,0 | 718,9 | 0,9965 |
|  | 40-59 | 449 | 342,0 | 10-878,4 | 39,0 | 777,2 |  |
|  | 60 ou mais | 114 | 339,3 | 6-870,4 | 36,8 | 787,2 |  |
| Plaquetas (μl) | 18-39 | 764 | 236.000 | 126.000-359.000 | 143.000 | 337.000 | 0,2158 |
|  | 40-59 | 464 | 230.000 | 138.000-364.000 | 148.000 | 338.000 |  |
|  | 60 ou mais | 125 | 223.000 | 138.000-353.000 | 146.000 | 333.000 |  |
| Volume plaquetário Médio (fL) | 18-39 | 633 | 10,4 | 7,6-13,2 | 8,2 | 12,6 | 0,2815 |
|  | 40-59 | 392 | 10,3 | 7,8-13,2 | 8,3 | 12,5 |  |
|  | 60 ou mais | 111 | 10,1 | 7,9-13,2 | 8,3 | 12,7 |  |

n: amostra. Min-Max: valor mínimo e valor máximo. LI: limite inferior (percentil 2,5). LS: Limite superior (percentil 97,5%). *Teste de Kruskal Wallis. a,b,c: letras iguais representam que não houve diferenças estatisticamente significativas entre os grupos (p> 0,05); a,b,c: letras diferentes representam que houve diferenças estatisticamente significativas entre os grupos (p≤ 0,05).

**Material Suplementar 7 – Intervalos de referência de hemograma séries vermelha e branca em adultos ≥ 18 anos segundo raça cor e sexo, Pesquisa Nacional de Saúde, Brasil, 2014-2015**

| **Exames** | **Sexo Masculino** | | | | | | **Sexo Feminino** | | | | | |  |
| --- | --- | --- | --- | --- | --- | --- | --- | --- | --- | --- | --- | --- | --- |
|  | **Raça** | **n** | **Mediana** | **min-max** | **LI** | **LS** | **Raça** | **n** | **Mediana** | **min-max** | **LI** | **LS** | |
| ***Série Vermelha*** | | | | | | | | | | | | | |
| Glóbulos vermelhos (milhões/mm^3^) | Branca | 522 | 5,1 | 3,6-6,0 | 4,4 | 5,8 | Branca | 578 | 4,5 | 3,8-6,0 | 4,0 | 5,2 | |
|  | Parda | 658 | 5,1 | 3,8-6,0 | 4,3 | 5,8 | Parda | 780 | 4,5 | 3,6-6,0 | 4,0 | 5,2 | |
|  | Preta | 100 | 5,1 | 4,0-5,2 | 4,3 | 5,8 | Preta | 98 | 4,5 | 3,8-5,9 | 3,9 | 5,4 | |
| Hemoglobina (g/dL) | Branca^*a^ | 525 | 15,1 | 13,0-17,5 | 13,3 | 16,8 | Branca^*a^ | 579 | 13,4 | 12,0-16,7 | 12,1 | 15,1 | |
|  | Parda^*b,c^ | 663 | 14,8 | 13,0-17,5 | 13,1 | 16,7 | Parda^*b^ | 780 | 13,2 | 12,0-17,3 | 12,0 | 15,0 | |
|  | Preta^*a,c^ | 102 | 15,0 | 13,1-17,3 | 13,2 | 17,7 | Preta^*b^ | 98 | 13,1 | 12,0-16,4 | 12,0 | 14,8 | |
| Hematócrito (%) | Branca | 520 | 46,0 | 38,3-53,2 | 40,5 | 52,2 | Branca | 578 | 41,0 | 35,5-51,6 | 36,8 | 47,7 | |
|  | Parda | 657 | 45,8 | 37,2-54,0 | 40,5 | 52,7 | Parda | 780 | 40,9 | 34,5-53,3 | 36,8 | 47,8 | |
|  | Preta | 101 | 45,6 | 39,2-52,9 | 39,8 | 52,3 | Preta | 98 | 40,6 | 36,3-50,0 | 36,6 | 45,2 | |
| Volume Corpuscular Médio (fL) | Branca | 501 | 90,5 | 79,1-102,8 | 82,8 | 99,7 | Branca^*a^ | 559 | 91,0 | 79,8-102,1 | 82,7 | 100,4 | |
|  | Parda | 634 | 90,3 | 78,8-102,8 | 81,8 | 101,4 | Parda^*a,b^ | 745 | 90,7 | 79,0-102,9 | 81,3 | 101,2 | |
|  | Preta | 100 | 88,5 | 78,7-100,7 | 80,8 | 100,5 | Preta^* b^ | 93 | 89,5 | 80,5-102,0 | 82,4 | 100,9 | |
| Hemoglobina Corpuscular Média (pg) | Branca^*a^ | 509 | 29,9 | 26,1-33,2 | 27,0 | 32,4 | Branca^*a^ | 565 | 29,8 | 26,0-33,2 | 27,0 | 32,3 | |
|  | Parda^*b^ | 641 | 29,7 | 26,0-33,2 | 26,5 | 32,2 | Parda^*b^ | 754 | 29,4 | 26,0-33,1 | 26,4 | 32,2 | |
|  | Preta^*b^ | 100 | 29,3 | 26,1-33,2 | 26,7 | 31,9 | Preta^*c^ | 94 | 28,9 | 26,0-32,9 | 26,3 | 31,8 | |
| Concentração de Hemoglobina Corpuscular Média (g/dL) | Branca^*a^ | 506 | 32,8 | 30,1-35,2 | 30,4 | 34,6 | Branca^*a^ | 541 | 32,7 | 30,1-35,2 | 30,4 | 34,2 | |
|  | Parda^*b^ | 620 | 32,6 | 30,1-35,0 | 30,3 | 34,4 | Parda^*b^ | 718 | 32,5 | 30,1-35,1 | 30,3 | 34,0 | |
|  | Preta^*b^ | 97 | 32,8 | 30,4-35,1 | 30,5 | 34,2 | Preta^*b^ | 90 | 32,4 | 30,3-34,1 | 30,5 | 33,7 | |
| Amplitude de distribuição dos eritrócitos (RDW) (%) | Branca | 511 | 13,4 | 11,9-15,6 | 12,2 | 15,1 | Branca | 557 | 13,4 | 11,5-15,6 | 12,1 | 15,2 | |
|  | Parda | 630 | 13,6 | 11,7-15,6 | 12,2 | 15,4 | Parda | 746 | 13,4 | 11,5-15,6 | 12,3 | 15,2 | |
|  | Preta | 100 | 13,3 | 11,8-15,6 | 12,2 | 15,5 | Preta | 92 | 13,5 | 11,9-15,6 | 12,2 | 15,6 | |
| ***Série Branca*** |  |  |  |  |  |  |  |  |  |  |  |  | |
| Glóbulos Brancos (mm^3^) | Branca | 479 | 5.800 | 1.900-10.900 | 3.100 | 9.800 | Branca^*a^ | 523 | 6.300 | 1.300-10.800 | 2.990 | 10.000 | |
|  | Parda | 595 | 6.000 | 2.100-10.630 | 2.700 | 9.600 | Parda^*b^ | 579 | 5.900 | 1.500-10.600 | 2.500 | 9.700 | |
|  | Preta | 93 | 6.200 | 2.600-9.900 | 3.200 | 9.200 | Preta^*b^ | 80 | 5.600 | 2.400-10.400 | 2.650 | 10.100 | |
| Neutrófilos Absolutos (mm^3^) | Branca | 476 | 3.123 | 400-7.101 | 954 | 6.106 | Branca^*a^ | 508 | 2.597 | 656-7067 | 966 | 6.401 | |
|  | Parda | 588 | 3.238 | 551-7.091 | 699 | 6.138 | Parda^*b^ | 670 | 3.129 | 194-7079 | 788 | 6.511 | |
|  | Preta | 91 | 3.006 | 741-6.232 | 791 | 5.888 | Preta^*b^ | 79 | 2.866 | 679-6072 | 715 | 6.027 | |
| Eosinófilos Absolutos (mm^3^) | Branca^*a^ | 442 | 151,2 | 0,0-741,0 | 17,1 | 648,0 | Branca^*a^ | 511 | 140,6 | 0,0-775,0 | 14,2 | 660,0 | |
|  | Parda^*b^ | 515 | 194,0 | 0,0-765,4 | 17,5 | 688,1 | Parda^*b,c^ | 630 | 150,0 | 0,0-768,0 | 9,3 | 694,4 | |
|  | Preta^*b^ | 86 | 230,1 | 16,5-763,8 | 38,5 | 678,5 | Preta^*a,c^ | 77 | 162,0 | 12,8-773,5 | 13,5 | 711,7 | |
| Basófilos Absolutos (mm^3^) | Branca | 462 | 23,4 | 0,0-88,9 | 0,0 | 71,8 | Branca | 520 | 22,0 | 0,0-96,6 | 0,0 | 68,3 | |
|  | Parda | 594 | 22,8 | 0,0-99,2 | 0,0 | 81,5 | Parda | 667 | 21,5 | 0,0-99,0 | 0,0 | 80,9 | |
|  | Preta | 90 | 27,6 | 0,0-80,4 | 0,0 | 66,8 | Preta | 81 | 24,0 | 0,0-99,0 | 0,0 | 96,8 | |
| Linfócitos Absolutos (mm^3^) | Branca | 463 | 2.033,5 | 420,0-3.843,0 | 775,2 | 3.557,8 | Branca | 522 | 2.032,8 | 342,0-3.686,0 | 764,4 | 3.360,0 | |
|  | Parda | 585 | 1.974,0 | 255,6-3.798,0 | 709,8 | 3.439,8 | Parda | 664 | 1.930,0 | 313,4-3.874,2 | 699,3 | 3.526,0 | |
|  | Preta | 91 | 2.007,5 | 577,2-3.726,8 | 744,8 | 3.492,8 | Preta | 81 | 2.128,0 | 369,0-3.634,0 | 612,3 | 3.631,0 | |
| Linfócitos  (%) | Branca | 462 | 35,1 | 10,3-62,3 | 14,6 | 58,5 | Branca | 523 | 32,8 | 5,5-62,3 | 13,1 | 56,1 | |
|  | Parda | 584 | 33,2 | 8,2-62,4 | 12,2 | 58,2 | Parda | 665 | 35,5 | 6,8-62,3 | 13,1 | 58,3 | |
|  | Preta | 91 | 33,0 | 11,4-60,6 | 17,0 | 59,3 | Preta | 81 | 35,3 | 9,0-61,3 | 12,6 | 58,7 | |
| Monócitos Absolutos (mm^3^) | Branca | 464 | 392,4 | 6,0-868,4 | 52,8 | 778,8 | Branca | 524 | 348,4 | 3,9-896,0 | 45,6 | 722,0 | |
|  | Parda | 582 | 400,4 | 3,1-900,6 | 52,2 | 782,0 | Parda | 671 | 343,2 | 0,0-886,5 | 39,0 | 788,8 | |
|  | Preta | 92 | 424,0 | 20,8-843,2 | 28,7 | 785,7 | Preta | 79 | 368,0 | 4,1-738,4 | 4,5 | 725,2 | |
| Plaquetas (μl) | Branca | 476 | 207.000 | 112.000-365.000 | 141.000 | 323.000 | Branca | 528 | 239.000 | 138.000-360.000 | 148.000 | 334.000 | |
|  | Parda | 600 | 213.000 | 105.000-358.000 | 143.000 | 355.000 | Parda | 714 | 226.000 | 126.000-364.000 | 143.000 | 341.000 | |
|  | Preta | 89 | 232.000 | 134.000-332.000 | 144.000 | 314.000 | Preta | 84 | 235.000 | 130.000-353.000 | 150.000 | 327.000 | |
| Volume plaquetário Médio (fL) | Branca | 389 | 10,2 | 7,7-13,0 | 8,2 | 12,4 | Branca^*a^ | 455 | 10,4 | 7,6-13,2 | 8,4 | 12,7 | |
|  | Parda | 482 | 10,1 | 7,5-13,2 | 8,2 | 12,6 | Parda^*b^ | 596 | 10,2 | 7,6-12,2 | 8,2 | 12,3 | |
|  | Preta | 75 | 10,0 | 7,6-12,8 | 8,1 | 12,4 | Preta^*a^ | 63 | 10,2 | 8,2-13,2 | 8,3 | 12,9 | |

n: amostra. Min-Max: valor mínimo e valor máximo. LI: limite inferior (percentil 2,5). LS: Limite superior (percentil 97,5%). *Valor p ≤ 0,05 pelo Teste de Kruskal Wallis. a,b,c,d: letras iguais representam que não houve diferenças estatisticamente significativas entre os grupos (p> 0,05); letras diferentes representam que houve diferenças estatisticamente significativas entre os grupos (p≤ 0,05).
