# Supplementary material for: Blood count reference intervals for the Brazilian adult population: National Health Survey
Source: Rev Bras Epidemiol. 2023 Apr 21;26(Suppl 1):e230004. doi: 10.1590/1980-549720230004.supl.1 (PMC10176730; doi:10.1590/1980-549720230004.supl.1)
Supplement: Translation

# Translation: Intervalos de referência de hemograma da população adulta brasileira: Pesquisa Nacional de Saúde

**DOI:** 10.1590/1980-549720230004.supl.1.1

**Authors:** Ana Carolina Micheletti Gomide Nogueira de Sá, Nydia Strachman Bacal, Crizian Saar Gomes, Tércia Moreira Ribeiro da Silva, Renata Patrícia Fonseca Gonçalves, Deborah Carvalho Malta

**Affiliations:** I Universidade Federal de Minas Gerais, Escola de Enfermagem, Programa de Pós-Graduação em Enfermagem – Belo Horizonte (MG), Brasil.; II Hospital Israelita Albert Einstein, Departamento de Patologia Clínica, Setor de Citometria de Fluxo – São Paulo (SP), Brasil.; III Universidade Federal de Minas Gerais, Faculdade de Medicina, Programa de Pós - Graduação em Saúde Pública – Belo Horizonte (MG), Brasil.; IV Universidade Federal de Minas Gerais, Escola de Enfermagem, Departamento de Enfermagem Materno Infantil e Saúde Pública – Belo Horizonte (MG), Brasil.; V Universidade Federal dos Vales do Jequitinhonha e Mucuri, Departamento de Enfermagem, Programa de Pós-Graduação em Ensino em Saúde – Diamantina (MG), Brasil.

**Keywords:** Inquéritos epidemiológicos, Valores de referência, Contagem de células sanguíneas, Leucócitos, Brasil

## Abstract

**Objetivo:**

Estimar os intervalos de referência (IR) de parâmetros de hemograma completo na população adulta brasileira.

**Métodos:**

Estudo transversal, com dados da Pesquisa Nacional de Saúde (PNS), entre 2014–2015. A amostra final constitui-se de 2.803 adultos. Para estabelecer os IR, aplicou-se critérios de exclusão, removeram-se *outliers* e foram feitos particionamentos por sexo, idade e raça/cor da pele. Adotou-se o método não paramétrico. As diferenças foram avaliadas pelos testes Mann Withney e Kruskal Wallis (p≤0,05).

**Resultados:**

Houve diferenças estatisticamente significativas nos IR segundo sexo para glóbulos vermelhos, hemoglobina, hematócrito, HCM, CHCM, eosinófilos, monócitos, neutrófilos absolutos e plaquetas (p≤0,05). Quando analisados por idade, houve diferenças nos IR de mulheres para hematócrito, VCM, glóbulos brancos e RDW, e nos homens em glóbulos vermelhos, glóbulos brancos, eosinófilos, volume plaquetário médios, VCM, RDW e HCM (p≤0,05). Para raça/cor, houve diferenças nos IR de hemoglobina, HCM, CHMC, glóbulos brancos e volume plaquetário médio, neutrófilos e eosinófilos absolutos (p≤0,05).

**Conclusão:**

As diferenças encontradas nos IR de alguns parâmetros de hemograma nos adultos brasileiros, reafirmam a importância de se ter padrões laboratoriais próprios de referência. Os resultados podem subsidiar a interpretação mais precisa dos exames, identificação adequada e a prevenção de doenças no Brasil.

## INTRODUÇÃO

Os intervalos de referência (IR) de hemograma (séries vermelha e branca) são importantes informações na prática clínica^1^ para a triagem de doadores de sangue, avaliação geral da saúde, estabelecimento eficaz de diagnóstico^2^, manejo e tratamento de doenças^1–3^.

IR fidedignos direcionam a identificação de doenças de importante magnitude, como anemia, infecções e neoplasias, e contribuem para o controle e prevenção^4^. A anemia representa um problema de saúde global; em 2019, correspondeu a 1,8 bilhões de casos prevalentes no mundo^5^, e no Brasil, de acordo com dados da Pesquisa Nacional de Saúde (PNS), entre 2014 e 2015, houve uma prevalência de 9,9% em adultos e idosos^6^. Globalmente, o câncer, em 2019, foi a segunda causa de óbitos (10.079.637), e no Brasil correspondeu a 266.034 mortes. As infecções do trato respiratório representaram a quarta causa de óbitos mundiais (2.493.199), sendo a terceira causa no Brasil (88.640)^7^.

Porém, estabelecer IR constitui-se um desafio em razão da exigência do rigor metodológico com a necessidade de amostra representativa da população; cuidados na coleta, transporte, análises bioquímicas e estatísticas^1,8^. Assim, determinar IR não é realidade de todos os países, restringindo-se aos que conduzem estudos populacionais^9^.

Ademais, os IR são influenciados por fatores, como raça, etnia, índice de massa corporal (IMC)^10^, ritmo circadiano, dieta, gravidez, ciclo menstrual^11,12^, menopausa^11^, atividade física, estresse, tabagismo e uso de medicações, bebidas alcoólicas ou cafeína^12^. Por isso, recomenda-se a determinação de IR para a população em que serão aplicados^10,13^, pois refletem a real condição de saúde^11^.

Mesmo sendo reconhecida a importância de se ter IR de próprios da população, no Brasil são adotados IR internacionais^1,12^. Até o momento, existe um único estudo, no qual foram calculados valores de referência para hemograma em adultos brasileiros com dados da PNS, pelo método paramétrico^1^.

A população tem influências nos valores de IR, por isso, avançar em métodos analíticos de cálculos pode minimizar tal efeito^2^. A aplicação de uma única abordagem para cálculo de IR pode levar à imprecisão, sendo recomendado testar outras metodologias^13^. Assim, torna-se importante a realização de estudos utilizando a mesma base de dados da PNS, em que se adotem diferentes métodos de determinação de IR.

Este estudo analisou pela primeira vez os IR de hemograma de adultos brasileiros com dados laboratoriais da PNS pelo método não-paramétrico, e conforme as recomendações da Diretriz C28-A3^14^, referência amplamente adotada pelos laboratórios^13^. Além disso, avançou ao ampliar os critérios de exclusão, incluir parâmetros de hemograma segundo raça/cor e nas análises utilizadas para particionamento em relação ao estudo previamente realizado no Brasil^1^.

Desta forma, o presente estudo teve como objetivo estimar IR de hemograma na população adulta brasileira.

## MÉTODOS

### Desenho do estudo

Estudo transversal, com dados da PNS, entre 2014 e 2015.

### Contexto e fonte de dados

A PNS é um inquérito de base domiciliar, realizada pelo Instituto Brasileiro de Geografia e Estatística (IBGE)^15,16^. Na PNS 2013 foram entrevistados 60.202 adultos. A coleta de exames foi planejada em subamostra de 25% dos setores censitários da pesquisa, e realizada entre 2014 e 2015, em 8.952 adultos^16^. As coletas de hemograma aconteceram a qualquer hora do dia em tubos com ácido etilenodiamino tetra-acético (EDTA). As amostras de sangue foram encaminhadas para os laboratórios de referência selecionados por atender o controle de qualidade do Ministério da Saúde. As amostras foram examinadas por analisador automático de células^16^.

Por conta do desenho complexo de amostragem da PNS e das probabilidades desiguais de seleção, foram calculadas ponderações por procedimentos de pós-estratificação. Os pesos amostrais foram adotados em todas as análises^16^. Maiores detalhamentos sobre o plano amostral da PNS, procedimentos de coleta, envio e armazenamento das amostras estão disponíveis em outras publicações^15,16^. A base de dados utilizada encontra-se disponível em: https://www.pns.icict.fiocruz.br/.

### Determinação dos intervalos de referência de hemograma

Para redução de fatores que podem influenciar nos IR^11^, e objetivando alcançar uma população saudável, foram aplicados critérios de exclusão embasados na literatura^1,8,14^ e ampliados os critérios adotados no estudo nacional^1^. Os critérios de exclusão utilizados, suas referências e pontos de cortes^17–21^ estão no Material Suplementar 1.

Para exclusão de *outlier*, utilizou-se inspeção visual e o método de Tukey. A identificação de *outliers* foi feita em intervalos interquartílico (IRQ), considerando-se o primeiro quartil (Q1) inferior e terceiro quartil (Q3) superior, segundo a fórmula: (Q1-(1,5xIRQ); Q3+(1,5xIRQ). Em níveis de <Q1-1,5 IQR e/ou >Q3+1,5 IQR, os *outliers* foram descartados^13^.

A amostra foi particionada segundo sexo, idade e raça/cor da pele, por meio de testes estatísticos^11,14^ e considerando-se as condições biológicas que influenciam os IR^11^.

Os IR foram estimados considerando 95% dos indivíduos saudáveis^9^, ligados aos percentis 2,5 e 97,5^14^. Foram utilizadas amostras acima de 120 indivíduos nos particionamentos por sexo e idade^14^.

### Participantes

Os participantes foram adultos a partir de 18 anos. Foi utilizada a base de dados PNS, composta por 8.952 indivíduos. Após procedimentos de exclusão e de retirada de *outliers*, a amostra final constituiu-se por 2.803 participantes.

### Variáveis

As variáveis incluídas foram: sociodemográficas e parâmetros de hemograma (séries vermelha e branca). A descrição completa das variáveis encontram-se no Material Suplementar 2.

### Análises estatísticas

As medianas foram calculadas para os limites de referência. O limite inferior (LI) foi ligado ao percentil 2,5 e o limite superior (LS), ao percentil 97,5 da distribuição da população de referência, segundo sexo, idade e raça/cor. Os IR foram estimados pelo método não-paramétrico, que ordena por tamanho as observações realizadas e as classifica considerando a menor r=1 até a maior r=n. O LI correspondeu a r=0,025 (n+1) e o LS, a observação da posição r=0,975 (n+1) do ranqueamento^14^.

Avaliaram-se a normalidade dos dados pelo teste de Shapiro Wilk e as diferenças pelos testes Mann Withney ou Kruskal Wallis, com pós-teste de Dun com correção de Bonferroni, com nível de significância de 5%.

As análises foram feitas no *Data Analysis and Statistical Software* (Stata), versão 14, e no *Software Package for the Social Science (SPSS),* versão 25.0, empregando-se o módulo *survey,* que considera os pesos de pós-estratificação.

### Aspectos éticos

A PNS foi aprovada pela Comissão Nacional de Ética em Pesquisa do Conselho Nacional de Saúde (parecer 328.159). A participação do adulto foi voluntária e a confidencialidade das informações, garantidas^15^.

## RESULTADOS

Os IR de glóbulos vermelhos (milhões/mm^3^), hemoglobina (g/dL) e hematócrito (%) foram maiores nos homens (4,3–5,7; mediana 5,1; 13,2–16,7; mediana 15,0; 40,4–52,4; mediana 45,8) que nas mulheres (4,0–5,2; mediana 4,1; 12,0–15,1; mediana 13,2; 36,8–47,7; mediana 41,0). Os LI de HCM (pg) foram mais elevados nos homens (26,7–32,3; mediana 29,8) que nas mulheres (26,5–32,3; mediana 29,6). No sexo masculino (30,3–34,4; mediana 32,8), os LS de CHCM (g/dL) também foram mais elevados que no feminino (30,4–34,1; mediana 32,6) (p≤0,05) (Tabela 1).

**Tabela 1:** Intervalos de referência de hemograma série vermelha em adultos ≥18 anos segundo sexo, Pesquisa Nacional de Saúde, Brasil, 2014–2015.

Exames	Sexo	n	Mediana	Min-Max	LI	LS	*p
Glóbulos vermelhos (milhões/mm^3^)	Masculino	1.299	5,1	3,6–6,0	4,3	5,8	<0,01
	Feminino	1.485	4,5	3,6–6,0	4,0	5,2	
Hemoglobina (g/dL)	Masculino	1.309	15,0	13,0–17,5	13,2	16,7	<0,01
	Feminino	1.486	13,2	12,0–17,3	12,0	15,1	
Hematócrito (%)	Masculino	1.297	45,8	37,2–54,0	40,4	52,4	<0,01
	Feminino	1.485	41,0	34,5–53,3	36,8	47,7	
Volume corpuscular Médio (fL)	Masculino	1.253	90,3	78,7–102,8	82,0	101,0	0,5156
	Feminino	1.425	90,7	79,0–102,9	81,9	100,9	
Hemoglobina corpuscular Média (pg)	Masculino	1.269	29,8	26,0–33,2	26,7	32,3	0,0031
	Feminino	1.442	29,6	26,0–33,2	26,5	32,3	
Concentração de hemoglobina corpuscular Média (g/dL)	Masculino	1.241	32,8	30,1–35,2	30,3	34,4	0,0006
	Feminino	1.376	32,6	30,1–35,2	30,4	34,5	
Amplitude de distribuição dos eritrócitos (RDW) (%)	Masculino	1.258	13,4	11,7–15,6	12,2	15,4	0,2229
	Feminino	1.422	13,4	11,5–15,6	12,2	15,3	

n: amostra; Min-Max: valor mínimo e valor máximo; LI: limite inferior (percentil 2,5); LS: limite superior (percentil 97,5).

* Teste Mann Withney.

IR de eosinófilos (mm^3^) e monócitos (mm^3^) foram superiores nos homens (17,5–676,4; mediana 167,5; 52,0–782,0; mediana 393,6), comparados às mulheres (11,7–671,6; mediana 150,2; 39,6–752,4; mediana 353,6). IR de plaquetas (μl) e neutrófilos (mm^3^) foram mais elevados em mulheres (145.000–337.000; mediana 234.000; 887,0–6.429,6; mediana 3.374,1) que em homens (143.000–315.000; mediana 210.000; 798,0–6.114,3; mediana 3.143,7) (p≤0,05) (Tabela 2).

**Tabela 2:** Intervalos de referência de hemograma série branca em adultos ≥ 18 anos segundo sexo, Pesquisa Nacional de Saúde, Brasil, 2014-2015.

Exames	Sexo	n	Mediana	Min-Max	LI	LS	p*
Glóbulos brancos (mm^3^)	Masculino	1.184	5.900	1.900–10.900	2.800	9.700	0,0977
	Feminino	1.308	6.120	1.300–10.800	2.600	9.900	
Neutrófilos absolutos (mm^3^)	Masculino	1.173	3.143,7	399,6–7101,0	798,0	6.114,3	0,0101
	Feminino	1.283	3.374,1	193,5–7078,5	887,0	6.429,6	
Eosinófilos absolutos (mm^3^)	Masculino	1.060	167,5	0,0–765,4	17,5	676,4	0,0006
	Feminino	1.246	150,2	0,0–775,0	11,7	671,6	
Basófilos absolutos (mm^3^)	Masculino	1.165	23,4	0,0–99,2	0,0	78,0	0,1459
	Feminino	1.295	22,2	0,0–99,0	0,0	80,5	
Linfócitos absolutos (mm^3^)	Masculino	1.158	1.996,9	255,6–3.843,0	717,0	3.511,2	0,8142
	Feminino	1.293	2.002,2	303,4–3.874,2	742,0	3.411,4	
Monócitos absolutos (mm^3^)	Masculino	1.157	393,6	3,1–900,6	52,0	782	<0,01
	Feminino	1.301	353,6	0,0–896,0	39,6	752,4	
Plaquetas (μl)	Masculino	1.183	210.000	105.000–365.000	143.000	315.000	<0,01
	Feminino	1.353	234.000	126.000–364.000	145.000	337.000	
Volume plaquetário médio (fL)	Masculino	962	10,1	7,5–13,2	8,2	12,6	0,0503
	Feminino	1.136	10,3	7,6–13,2	8,3	12,6	

n: amostra; Min-Max: valor mínimo e valor máximo; LI: limite inferior (percentil 2,5); LS: limite superior (percentil 97,5).

* Teste Mann Withney.

Nos homens, os IR de glóbulos vermelhos (milhões/mm^3^) foram mais elevados entre 18 e 39 anos (4,4–5,8; mediana 5,1), comparando-se com as faixas etárias de 40 a 59 anos (4,3–5,7; mediana 5,0) e 60 anos ou mais (4,2–5,8; mediana 4,8). IR de VCM (fL) e HCM (pg) foram mais baixos nos homens entre 18 e 39 anos (81,8–100,4; mediana 89,4; 26,4–32,0; mediana 29,5) e de 40 a 59 anos (82,6–101,2; mediana 91,4; 26,0–32,2; mediana 30,0) do que nos com 60 anos ou mais (83,9–102,0; mediana 92,7; 27,2–38,2; mediana 30,7) (p≤0,05). A mediana e LS de RDW (%) foram mais elevados em homens a partir de 60 anos (12,2–15,5; mediana 13,5), do que nos com 18 a 39 anos (12,4–15,3; mediana 13,3) e 40 a 59 anos (12,3–15,3; mediana 13,3) (p≤0,05) (Material Suplementar 3).

Nas mulheres, houve diferenças para os LI e LS, e a mediana foi mais baixa para hematócrito (%) entre 18 e 39 anos (36,9–47,3; mediana 40,7), quando se comparou com 40 a 59 anos (36,6–48,4; mediana 41,3) e 60 anos ou mais (36,5–47,1; mediana 41,2). IR de VCM (fL) foram mais baixos nas mulheres entre 18 e 39 anos (81,6–100,9; mediana 90,5) do que aos 40 a 59 anos (82,2–100,7; mediana 91,2). Foram mais baixos os LI e mediana de RDW (%) nas mulheres entre 18 e 39 anos (12,1–15,2; mediana 13,4) do que nas de 40 a 59 anos (12,3–15,4; mediana 13,5) e 60 anos ou mais (12,3–15,2; mediana 13,7) (p≤0,05) (Material Suplementar 4).

Homens tiveram maiores contagens de glóbulos brancos (mm^3^) entre 18 e 39 anos (2.970–9.990; mediana 6.000), comparado com 60 anos ou mais (2.600–9.400; mediana 5.640). LI e medianas de eosinófilos (mm^3^) foram mais elevados nos homens entre 18 e 39 anos (26,1–661,2; mediana 190,1), comparando-se com 40 a 59 anos (16,8–679,8; mediana 141,6 mm^3^). IR de plaquetas (μl) foram mais baixos nos homens com o aumento da idade (18 a 39 anos: 144.000–314.000; mediana 215.000; 40 a 59 anos: 143.000–322.000; mediana 215.000; 60 anos ou mais: 138.000–306.000; mediana 203.000; p≤0,05) (Material Suplementar 5).

Nas mulheres, houve diferenças para IR de glóbulos brancos (mm^3^); a mediana e os LI foram mais proeminentes entre 18 e 39 anos (2.600-10.000; mediana 6.300) e de 40 a 59 anos (2.800–9.800; mediana 5.800) do que aos 60 anos ou mais (2.000–9.800; mediana 5.500 mm^3^) (p≤0,05) (Material Suplementar 6).

IR de hemoglobina (g/dL) e de HCM (pg) foram mais elevados nos homens brancos (13,3–16,8; mediana 15,1; 27,0–32,4; mediana 29,9) do que nos pardos (13,1–17,3; mediana 14,8; 26,5–32,2; mediana 29,7). As medianas de HCM foram mais baixas em homens pretos (29,3 pg) do que nos brancos (29,9 pg). O LS e a mediana de CHCM (g/dL) foram levemente mais elevados em homens brancos (30,4–34,6; mediana 32,8) que nos pardos (30,3–34,4; mediana 32,6) e pretos (30,5–34,3; mediana 32,8) (p≤0,05). IR de eosinófilos (mm^3^) foram mais baixos nos homens brancos (17,1–648,0; mediana 151,2) do que nos pretos (38,5–678,5; mediana 230,1) e pardos (17,5–688,1; mediana 194,0) (p≤0,05) (Material Suplementar 7).

IR de hemoglobina (g/dL) e HCM (pg) foram mais elevados nas mulheres de raça/cor branca (12,1–15,1; mediana 13,4; 27,0–32,3; mediana 29,8) do que nas pardas (12,0–15,0; mediana 13,2; 26,4–32,2; mediana 29,4) e pretas (12,0–14,8; mediana 13,1; 26,3–31,8; mediana 28,9). A mediana e LI de VCM (fL) foi mais proeminente nas mulheres brancas (82,7–100,4; mediana 91,0) do que nas pretas (82,4–100,9; mediana 89,5). O LS de CHCM (g/dL) foi mais elevado nas mulheres brancas (30,4–34,2; mediana 32,7) que nas pardas (30,3–34,0; mediana 32,5) e pretas (30,5–33,7; mediana 32,4). Mulheres brancas (2.900–10.000; mediana 6.300) apresentaram maiores valores de mediana e de LI para glóbulos brancos (mm^3^) do que as pardas (2.500–9.700; mediana 5.900) e pretas (2.650–10.100; mediana 5.600). Nas mulheres pardas (mediana 3.129) e pretas (mediana 2.866), as medianas de neutrófilos (mm^3^) foram mais elevadas que nas brancas (mediana 2.597). Nas mulheres pardas (9,3–694,4; mediana 150,0), foram mais elevados a mediana e o LS de eosinófilos (mm^3^) do que nas brancas (14,2–660,0; mediana 140,6). A mediana e o LI de volume plaquetário médio (fL) foi mais elevado nas mulheres brancas (8,4–12,7; mediana 10,4) que nas pardas (8,2–12,3; mediana 10,2) e pretas (8,3–12,9; mediana 10,2) (p≤0,05) (Material Suplementar 7).

## DISCUSSÃO

Neste estudo foram estimados IR de hemograma de adultos brasileiros pelos exames da PNS, pelo método não-paramétrico. Foram observadas diferenças estatisticamente significativas para alguns componentes do hemograma séries vermelha e branca quando analisados segundo sexo, idade e raça/cor. Os IR foram calculados por metodologia até então não testada, seguindo recomendações da literatura, no intuito de se obter valores cada vez mais acurados e confiáveis^13^. Por conseguinte, diferenciou-se do único estudo nacional existente em que foram calculados valores de referência para adultos brasileiros, ao se utilizar abordagem não paramétrica e também pelos testes aplicados para estratificação da amostra, aplicação do método de Tukey para retirada de *outlier* e a ampliação dos critérios de exclusão do estudo de Rosenfeld et al.^1^.

A metodologia não-paramétrica aqui adotada está em consonância com estudos realizados em Gana^3^, Canadá^8^, Índia^9^, Kênia^10^ e Coreia^22^. Esse método é recomendado em razão de muitos analitos não apresentarem distribuição normal, e por ser mais simples, dependente apenas das classificações dos dados de referência dispostos em ordem crescente de tamanho^14^. A literatura descreve que os resultados encontrados nos métodos paramétrico e não-paramétrico costumam ser semelhantes^14^. Como pode se observar pelos valores de mediana encontrados neste estudo segundo sexo e nos valores das médias identificadas no estudo nacional para glóbulos vermelhos e brancos foram de 5,0 milhões/mm^3^ e 6.142 mm^3^ em homens e 4,5 milhões/mm^3^ e 6.426 mm^3^ em mulheres, respectivamente^1^.

Para seleção dos indivíduos saudáveis, as exclusões foram definidas conforme Diretriz C28-A3^14^ e embasadas em estudos^1,8–10,23^. Os procedimentos aqui adotados para remoção de *outliers* foram utilizados em estudos no Canadá^8^ e Omã^24^. O método de Tukey foi utilizado por ser mais útil e indicado na presença de mais de um *outlier* ^13^, o que ocorreu neste estudo, e a inspeção visual por ser considerada eficaz^13^.

Considerando o particionamento como uma ferramenta de poder de diagnóstico dos IR^7^, nesta investigação foram empregados testes estatísticos para verificar a sua necessidade, assim como em outros estudos^22–25^. Os particionamentos adotados constam na Diretriz C28-A3^15^, e também considerou-se as alterações fisiológicas da fase adulta^11^. No cálculo de IR, os aspectos fisiológicos são tão importantes quanto os estatísticos^11,14^, pois IR diferem-se em crianças, adultos, idosos, homens e nas mulheres; na puberdade, gravidez e menopausa, por alterações orgânicas no decorrer da vida^11^.

Os IR mais elevados de hemácias, hemoglobina e hematócrito identificados nos homens, comparados às mulheres, e mais elevados de plaquetas em mulheres que nos homens, também foram encontrados em Gana^3^, Omã^24^ e Brasil^1^. Neste estudo, assim como em uma investigação realizada no Marrocos^25^, foram observados IR mais elevados de HCM e CHCM nos homens em relação às mulheres. São documentadas diferenças entre sexos para eritrócitos, hemoglobina, hematócrito, HCM, CHCM, plaquetas e volume plaquetário^1^, que podem ser justificadas pelo efeito da menstruação e a maior demanda por ferro^3,23^, influências hormonais do androgênio^1,3,8^ e a forma como a eritropoiese e a megacariopoiese são reguladas nos homens e mulheres^3^.

Nossos achados concordam com estudos do Brasil^1^, Canadá^8^, Coreia^23^ e Marrocos^25^, em que os níveis de neutrófilos foram mais baixos nos homens que nas mulheres, possivelmente pelos impactos relacionados ao desenvolvimento sexual na imunidade, em que o estrogênio estimula resposta imunológica, e alguns hormônios andrógenos, como a testosterona, suprimem a resposta à infecção, sendo os níveis de neutrófilos mais elevados em mulheres durante a puberdade e na idade adulta^8,23^.

Estudos realizados no Brasil^1^, Canadá^8^ e China^22^, encontraram diferenças nos IR de monócitos segundo sexo^8,22^, sendo mais elevados nos homens, em consonância com os nossos resultados. Sabe-se que homens podem apresentar contagens mais elevadas de monócitos que mulheres, contudo os aspectos fisiológicos e clínicos ainda carecem de elucidação^8^. Os IR mais elevados de eosinófilos em homens do que nas mulheres também foram encontrados em estudos na Coreia^23^, Marrocos^25^, China^22^, Gana^3^ e Brasil^1^. Contudo, são necessárias pesquisas que investiguem os motivos das flutuações biológicas desses parâmetros leucocitários em populações específicas de distintas localidades segundo sexos^9^.

As diferenças sutis encontradas nos IR de hematócrito de mulheres, sendo mais elevados a partir dos 40 anos, em relação a 18 a 39 anos, se justificam pela variabilidade desse parâmetro que é semelhante ao longo da vida^8^. O aumento ligeiro de hematócrito com a idade foi encontrado em adultas chinesas. Esse achado é consistente com os níveis mais baixos antes da menopausa e mais altos após esse período^2^. Os valores de glóbulos vermelhos tenderam a diminuir com a idade nos homens, como em adultos chineses^2^. A diminuição pode ser decorrente da perda gradual de andrógeno^2^. Os IR mais reduzidos a partir de 60 anos nos homens podem estar relacionados à deficiência nutricional, malignidade oculta e anemia^2^.

Para IR de glóbulos brancos, os resultados encontrados se assemelham com outros estudos^23,24^, em que observaram-se um declínio com o aumento da idade^8^. Os achados reafirmam que poderiam ser realizadas duas partições por idade para esse parâmetro, como no estudo brasileiro^1^, pois não houve diferenças entre as duas faixas etárias de 18 a 39 e 40 a 59 anos. As maiores contagens de leucócitos em adultos jovens de ambos os sexos, quando comparadas às menores contagens em idosos, refletem o desenvolvimento da resposta imune adquirida e adaptativa à medida que o sistema imunológico está mais exposto a patógenos e antígenos no ambiente^8,23^.

Neste estudo, o ligeiro aumento dos LS de eosinófilos com a idade nos homens pode estar relacionado a infecções crônicas em idosos que não foram excluídos^2^. Esse achado esteve presente em adultos chineses de ambos os sexos^2^. As diferenças nos valores de eosinófilos podem ser atribuídas às doenças alérgicas e parasitárias nos adultos aparentemente saudáveis^2^.

Estudos no Canadá^8^ e no Brasil^1^ também identificaram o aumento ligeiro de VCM e RDW ao longo da vida, com pequenas mudanças no início da idade adulta, permanecendo constantes após essa fase^8^. Estudo na China identificou que idosos de ambos os sexos apresentaram maiores IR de RDW^23^. O VCM e o RDW fornecem uma classificação dos eritrócitos com base no tamanho e distribuição^4^. Para valores VCM, o LI é de aproximadamente 70 fL somado a idade (em anos), e o LS pode ser obtido adicionando 0,6 fL por ano a 84 fL para além do primeiro ano de vida, até se atingir aproximadamente 96 fL em adultos^4^, consistente com os achados desse estudo. O RDW é útil na identificação de deficiência de ferro, traço de β-talassemia, processos inflamatórios e infecção crônica^4^. Também é preditor de mortalidade, e sua elevação com a idade está relacionada a alterações orgânicas do envelhecimento e a doenças crônicas nessa fase^1^.

Neste estudo, as diferenças significativas dos valores de HCM com a idade em homens, sendo mais elevado em idosos, foi identificada em estudo prévio^1^. Destaca-se que os IR para todas as faixas etárias em homens brasileiros foram mais baixos que a classificação hematrimétrica internacional (27 a 33,7 pg)^2^. A literatura evidencia variações regionais para HCM; em chineses, IR não diferiram com idade e sexo^2^, já em homens marroquinos houve diferenças^25^. Embora nosso estudo tenha encontrado diferenças em homens, a classificação internacional abrange os mesmos valores de IR para HCM segundo sexo e idade para adultos^2^. Nesse sentido, novas investigações são desejáveis para esclarecer as implicações dessas diferenças na prática clínica.

A menor contagem de plaquetas identificada em homens idosos brasileiros está em conformidade com um estudo no Canadá^8^ e foi encontrada em homens após os 40 anos no Irã^9^. Possível justificativa é o declínio gradual de trombopoietina, hormônio que regula a produção de plaquetas nos adultos. A ocorrência de trombocitopenia é mais comum em homens do que nas mulheres, e mais frequente em idosos^8^.

Neste estudo, foram estabelecidos IR para 16 parâmetros de hemograma segundo raça/cor. Em pesquisa prévia, foram determinados IR de cinco parâmetros, sendo os valores encontrados próximos aos descritos^1^. Apesar de discretas, as diferenças encontradas segundo raça/cor para IR de hemoglobina, HCM, CHMC, glóbulos brancos e volume plaquetário médio, neutrófilos e eosinófilos, apoiam a necessidade de estabelecer IR para brasileiros. Diferenças nos IR de hemograma foram encontradas em populações de outros países^1–3,8,10,24^, reforçando a importância de se considerar as influências geográficas e étnico-raciais, pois podem ter relação com fatores genéticos, nutricionais, socioeconômicos, culturais, estilos de vida, exposição a alérgenos, infecções e cargas parasitárias nas distintas localidades^3^.

Limitações, como a possibilidade de inclusão de doentes sem diagnóstico prévio e a não obtenção de amostras com 120 indivíduos para alguns estratos de raça/cor, devem ser consideradas. Contudo, em virtude da representatividade da amostra, destaca-se que este estudo se aproxima da realidade das condições de saúde da população brasileira. Ainda, a literatura documenta que é possível estabelecer IR de 95% utilizando até 39 amostras^13^. Quanto às perdas atribuídas aos procedimentos de exclusão e remoção de *outliers*, trata-se de um viés conservador na prática clínica, pois foi possível estimar os IR de hemograma para adultos brasileiros e predizer que os valores encontrados se aproximaram do estudo nacional prévio^1^, levando-se em consideração as recomendações de se testar outras abordagens metodológicas^13^. Os IR aqui encontrados conferem confiabilidade e permite a generalização dos achados de forma relativamente segura. As diferenças encontradas em alguns parâmetros de hemograma em adultos brasileiros segundo sexo, idade e raça/cor mostram que existe a necessidade de se estabelecer IR adequados à população. Os resultados evidenciam as influências étnico-raciais nos IR e podem subsidiar a identificação e prevenção de doenças, bem como pesquisas futuras para validação de IR da população brasileira, contribuindo com melhor interpretação, precisão diagnóstica e qualidade dos cuidados e tratamento ofertados.
