# Supplementary material for: Erratum
Source: Rev Bras Epidemiol. 2024 Sep 30;27:e20230004erratum. doi: 10.1590/1980-549720230004.supl.1erratum (PMC11476858; doi:10.1590/1980-549720230004.supl.1erratum)
Supplement: Related texts

# Correction: Errata

**DOI:** 10.1590/1980-549720230004.supl.1.1erratum

No artigo "**Intervalos de referência de hemograma da população adulta brasileira: Pesquisa Nacional de Saúde**", DOI: https://doi.org/10.1590/1980-549720230004.supl.1.1, publicado no periódico Rev Bras Epidemiol. 2023; 26(Suppl 1): e230004.supl.1:

**Na página 1 foi incluído:**

EDITORA CIENTÍFICA: Márcia Furquim de Almeida http://orcid.org/0000-0003-0052-1888
